# Motion of droplets into hydrophobic parallel plates

**DOI:** 10.1039/c9ra05135j

**Published:** 2019-10-10

**Authors:** Xiongheng Bian, Haibo Huang, Liguo Chen

**Affiliations:** Robotics & Microsystem Center, Collaborative Innovation Center of Suzhou Nano Science and Technology, Soochow University Suzhou 215123 China hbhuang@suda.edu.cn chenliguo@suda.edu.cn

## Abstract

Due to the superior operability and good anti-interference, the prospect of controlling microdroplets using a parallel plate structure (PPS) is very promising. However, in practical applications, droplets in such structures are often affected by various factors, resulting in deformation, evaporation, stress rupture and other phenomena, leading to equipment failure. Therefore, how to simply and effectively transfer liquid droplets to PPS to maintain the stable and efficient operation of the system has become an urgent problem to be solved. In this paper, a simple and effective ratchet-like strategy (relaxing and squeezing actions) is introduced to transfer droplets. To analyze the mechanism of the strategy and optimize the control, we conduct this study from three aspects. First, the droplet movement trend is obtained by analyzing the pressure between SPS and PPS. Second, the reasons why the droplet can achieve this inward motion are investigated. Through theoretical analysis, which is also proven by simulations and experiments, we creatively put forward that the asymmetric change of the contact angle (CA) induced by the asymmetric structure is the fundamental cause of this kind of motion. Due to the asymmetric change of the contact angle, the CA in the PPS will reach the advancing angle first in the squeezing process, and the CA in the SPS will reach the receding angle first in the relaxing process, thus causing the inward movement of the droplet. Third, to optimize this strategy, the effects of the following governing parameters are researched individually based on the corresponding simulations and experiments: the control parameters (the initial gap width of the PPS *H*_0_ and the amount of squeezing and relaxing of Δ*H*) and the thickness of the top plate. Subsequently, an optimized ratchet-like cycle is achieved. In summary, these findings not only provide a new method by which to realize the movement of droplets toward hydrophobic PPSs but also creatively point out the cause of the ratchet strategy, which can be applied in many microfluidics fields.

## Abstract 2

A simple and effective ratchet-like strategy is introduced to transfer droplets. We creatively put forward that the asymmetric change of the contact angle induced by the asymmetric structure is the fundamental cause of this kind of motion.

## Graphical abstract

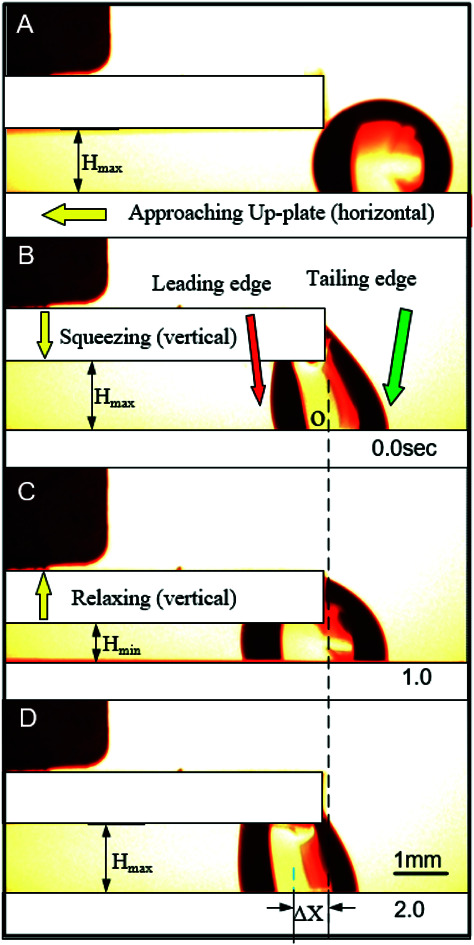

## 1. Introduction

In recent years, the control of droplet motion has been extensively discussed as one of the most important fluid control strategies. Various droplet driving methods have been thoroughly studied,^1^ among which the most common methods include surface temperature changes, surface chemical properties,^2–4^ hydrophilic and hydrophobic surface structures,^5–7^ electric field application,^8–11^ acidic and alkaline (pH) environments,^12^ mechanical vibration,^13–18^*etc.* Droplet operation is widely used in microfluidic chips,^19–21^ integrated circuits,^22^ industrial experimental equipment,^23^ liquid collection^24^ and other fields. Hydrophobic parallel plate structure (PPS) is widely used in various applications due to its good maneuverability and good anti-interference. As is known, although there are many ways to realize the unidirectional movement of liquid droplets, there are few ways to realize the transport of droplets into a hydrophobic PPS. Generally, an electrowetting force is always used to achieve this. However, this method often requires high voltages,^25,26^ which can damage microfluidics chips and has an effect on biochemical substances in the droplets. A novel concise method is needed to solve this problem; thus, the ratchet-like strategy is first introduced.

It is well known that droplets on wetting gradient surfaces can move along the design direction, and repeated squeeze-release droplets can reduce the hysteresis caused by contact angle hysteresis (CAH).^27^ In previous studies, the ratchet mechanism has mainly been applied to uniform structures (SPS, PPS or non-parallel plate structures). The case of nonparallel plates has been most extensively studied. According to research,^28,29^ droplets sandwiched between two non-parallel plates with a hydrophilic surface were observed to move toward the tip due to relaxing and squeezing the droplets. This phenomenon has been speculated to be mainly caused by the asymmetric depinning (AD) of droplets, which means that the trailing edge remains pinned as the leading edge advances during the squeezing process and that the leading edge remains pinned as the trailing edge recedes during the relaxing process.^28,30–33^ Later research found that the droplet is transported not only by the AD but also by symmetric depinning (SD), which means that both the leading and trailing edges are advancing or receding.^31^ In fact, the efficiency of the SD model is much lower than that of the AD model. If the SD model is included in the transport process, the movement efficiency of the liquid bridge will be reduced.^30^ Ataei *et al.^30,38^* optimized the ratchet parameters to achieve a more efficient ratchet cycle by decreasing the SD model. Wang *et al.*^34^ demonstrated that hydrophobic saw-tooth plates could improve the transport efficiency substantially by obstructing the inefficient SD model. In addition to the uniform structure, Huang *et al.*^31^ proposed a parallel–nonparallel (P–NP) combinative structure for immersion lithography; however, it cannot be used in hydrophobic situations. How to transport droplets from an SPS into a PPS, which can be widely used in microfluidics applications, has not been investigated.

In this paper, a ratchet-like strategy is introduced to move a droplet into hydrophobic parallel plates. First, we demonstrate the process of the droplets entering the hydrophobic PPS using a squeezing and relaxing (ratchet-like) strategy. Second, the pressure on the droplet pinned between the PPS and SPS is analyzed and simulated in detail. On this basis, the reason why the droplet can enter the PPS through the ratchet-like strategy is given theoretically and verified by experiments and simulations. Finally, the control parameters are analyzed and optimized, and a good performance is obtained, which is verified by simulations and experiments.

## 2. A novel strategy to move droplets into hydrophobic PPS

**Fig. 1:**
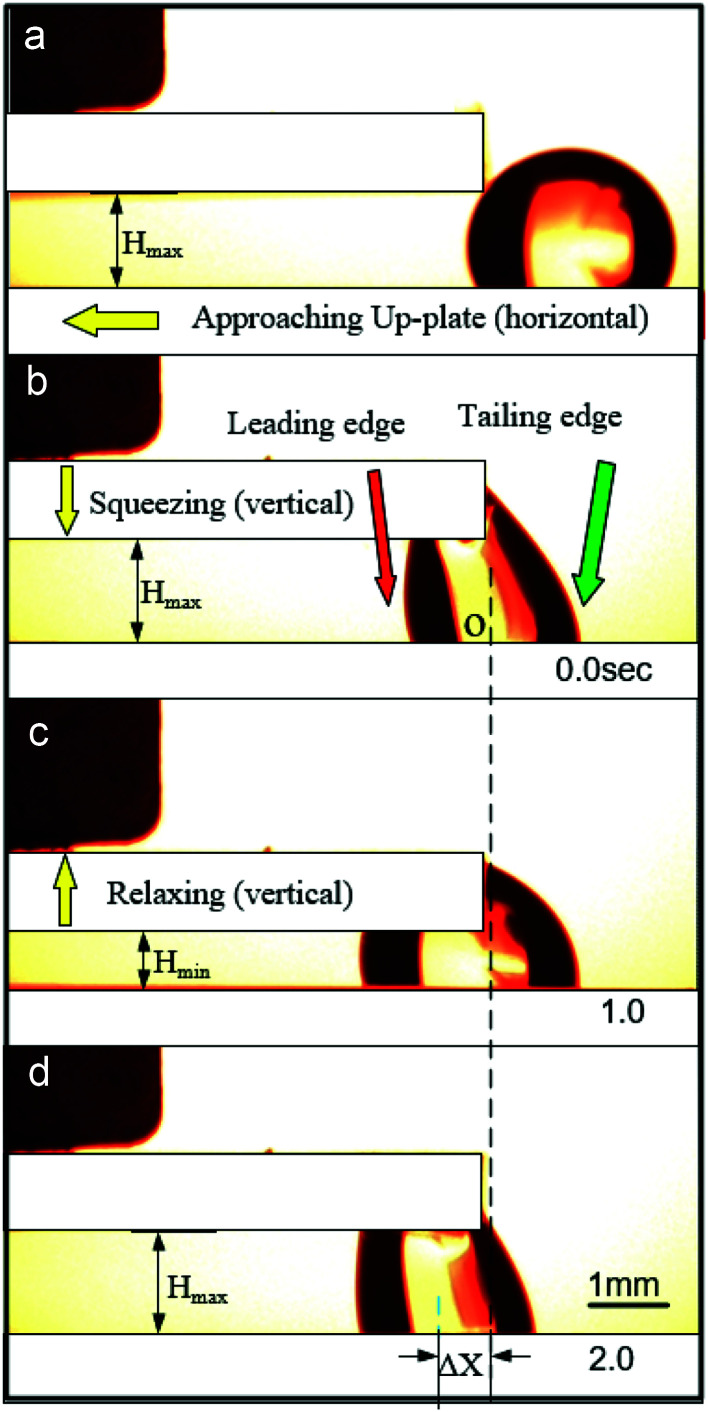
(a) Initial position of the droplet before bridge formation. (b) Initial position of the liquid bridge after bridge formation. The gap width is represented by *H*_max_. (c) The bridge is squeezed for Δ*H*, and the gap width is represented by *H*_min_. (d) Subsequently, the squeezed liquid bridge was relaxed for Δ*H* to *H*_max_ to finish the ratchet-like cycle.

This novel strategy was shown in Fig. 1. The entire control system was placed horizontally, with the top and bottom plates parallel to each other. Droplets were placed between the SPS and PPS. The upper and lower interfaces of the SPS and PPS were hydrophobic. The boundary of the liquid droplet in the PPS was called the leading edge, and the boundary in the SPS was called the trailing edge. The initial contact angle (CA) was similar to the advancing contact angle (*α*_a_). Meanwhile, the top plate was placed over the bottom plate with a set initial gap width (*H*_max_). The bottom plate was moved horizontally toward the top plate at a low speed (0.05 mm s^−1^); therefore, the droplet was considered quasi-static. As soon as the top plate touched the droplet, the bottom plate movement was stopped immediately (Fig. 1a). Then, a liquid bridge was formed between the PPS and SPS (Fig. 1b). Subsequently, a slow downward movement of the top plate was carried out (0.4 mm s^−1^) to squeeze the bridge for a distance of Δ*H* (Fig. 1b and c), and then the liquid bridge was relaxed the same amount (Fig. 1c and d). Afterward, the droplet could move toward this hydrophobic PPS. The midpoint of the leading and trailing edges (point O in Fig. 1b) was used as the reference point to measure the distance of the liquid bridge movement in the horizontal direction. This liquid bridge movement was marked as Δ*X* (as in Fig. 1d), and the direction toward the PPS was recognized as positive.

## 3. Analysis of the pressure applied on the droplets pinned between the SPS and PPS

**Fig. 2:**
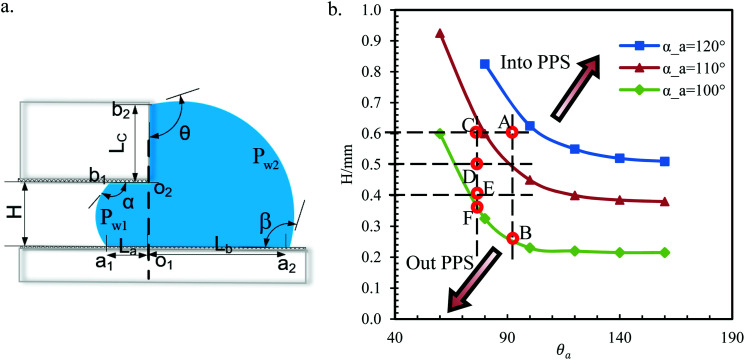
(a) Schematic diagram of the droplet pinned between the SPS and PPS. (b) Simulations to determine exactly how the pressure difference (Δ*P*) is affected by various parameters (*α*_a_, *θ*_a_ and *H*).

We first analyzed the pressure on the droplets pinned between the PPS and SPS. As shown in Fig. 2a, the variables on the profile are defined, and *H* and *α* are the width of the gap and the CA in the PPS, respectively. The intersections of the leading edge with the top and bottom plate are labeled *b*_1_ and *a*_1_, respectively, and those of the trailing edge with the top and bottom plate are labeled *b*_2_ and *a*_2_, respectively. *o*_2_ represents the intersection point between the top plate side surface and the bottom plate interface, and *o*_1_ represents the intersection point between the vertical extension line of the top plate side surface and the bottom plate, which also divides the bottom plate into two SPS and PPS zones. *L*_a_ represents the length of the droplet in the PPS, *L*_b_ represents the length of the droplet in the SPS (*o*_1_*a*_2_), and *L*_c_ represents the length of the droplet on the plate (*o*_2_*b*_2_). *θ* and *β* represent the wetting angles between the droplet and the top plate side surface 7 and the bottom interface of the SPS, respectively. Then, the pressure in the PPS, which is labeled *P*_w1_, can be written as follows:1
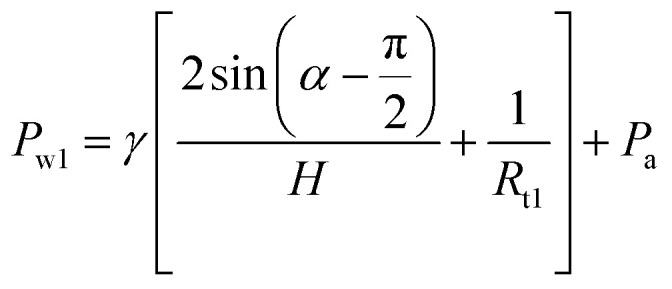


Similarly, the surface tension of the SPS, which is labeled *P*_w2_, can be calculated by the following formula:2
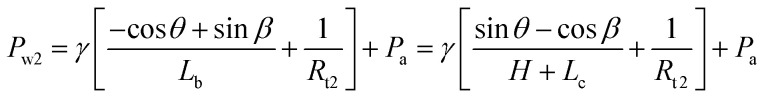
where *P*_a_ is the atmospheric pressure and *R*_t1_ and *R*_t2_ are the horizontal curvatures of the pinned droplet in the PPS and SPS, respectively. Therefore, the pressure difference between the PPS and SPS (Δ*P* = *P*_w2_ − *P*_w1_) can be represented as:3
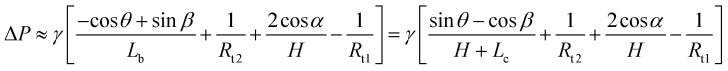


From the equation, we find that when the droplets are on the hydrophobic surface (*α* > 90°), *α* and Δ*P* are negatively correlated—the smaller is *α*, the greater is Δ*P*. *H* is positively correlated to Δ*P*; thus, the larger is *H*, the larger is Δ*P*. However, because of the coupling of *θ* and *β* and their correlation with *L*_b_ and *L*_c_, it is difficult to analyze the relationship between *θ*, *β* and Δ*P* directly. Therefore, the such relationships were also studied based on the simulation results.

### 3.1 Analysis of the Laplace pressure based on simulations

Without considering CAH (*α*_a_ = *α*_r_, *θ*_a_ = *θ*_r_), we discussed the influence of *α*_a_, *θ*_a_ and *H* through simulations. During the simulations, the volume of the droplet is set to 0.69 μL. Since the gap width is related to the volume of the droplet, to calculate the minimum gap width, the parameter 
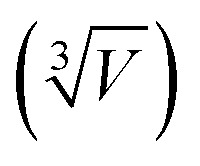
 is multiplied. The results are shown in Fig. 2b, in which the curves represent the situation when Δ*P* = 0. The upper and lower parts of the curves indicate that the droplets tend to move inward or outward, respectively. The pressure difference is related to the vertical range between the actual parameters and the relevant curves. For example, if the actual experimental parameters are *α*_a_ = 100°, *θ*_a_ = 90° and *H* = 0.6 mm (point A), then the corresponding point on the curve is point B. Therefore, Δ*P* is positively related to the length of segment AB. According to these results, three conclusions can be drawn. First, a larger gap width is beneficial to droplet inward motion. Second, for a constant *α*_a_, a larger *θ*_a_ promotes inward motion (a bigger Δ*P*). But as *θ*_a_ increases, the change rate of the gap width (*H*) *versus θ*_a_ curves decreases, which illustrates the weaker influence of *θ*_a_ on Δ*P*. Third, contrary to the effect of *θ*_a_, as *α*_a_ increases, a larger gap width is needed to achieve inward motion, which also means that a larger *α*_a_ hinders the inward motion.

### 3.2 Hysteresis force induced by CAH

The influence of CAH on droplet motion is mainly reflected by the influence of CA in eqn (1) and (2). Because of CAH, contact angles *α*, *θ* and *β* are no longer fixed values but are ranges of quantities, *i.e.*, *α*, *β* ∈ (*α*_r_, *α*_a_) and *θ* ∈ (*θ*_r_, *θ*_a_), which are the reasons why CAH hinders the inward motion. For example, when *α* = *α*_a_ = 100°, the droplet tends to move inward, that is to say, *P*_w1_ < *P*_w2_ without considering CAH. But due to the function of CAH, although *α* is still close to *α*_a_ (*P*_w1_ remains roughly the same), *β* can be smaller than *α*_a_, and *θ* can also be smaller than *θ*_a_. Therefore, according to eqn (2), *P*_w2_ can be smaller. If *P*_w2_ is no longer larger than *P*_w1_, then there won't be inward motion. From this result, it can be found that a larger CAH (*α*_CAH_ and *θ*_CAH_) can cause a larger hysteresis force to hinder the motion into the PPS.

## 4. Analysis of droplet transport into the PPS by the ratchet-like strategy

### 4.1 Experiment and simulation fundamentals

#### 4.1.1 Experiment fundamentals

In the following experiments, glass was chosen as the plate material, and the surfaces were cleaned ultrasonically for 10 minutes. Subsequently, the upper surface of the bottom plate and the lower surface of the top plate were treated with hydrophobic materials. Upon placing droplets on different surfaces, we recorded and measured their actual contact angles using a CCD camera. The first type of material was Teflon-AF1600 (DuPont). The receding and advancing contact angles of the Teflon-coated sample were approximately 99° and 119°, respectively, with an error of 3°. The other type was hydrophobic-treated SiO_2_, which had an advancing angle of 100° and a receding angle of 80°, with an error of 3°. The receding and advancing contact angles of the nontreated sample were approximately 45° and 75°, respectively, with an error of 3°. The motion of the top plate was precisely controlled and located using a micro-motion platform. To ensure that the droplet was in the quasi-static state, the movement speed was set to 0.4 mm s^−1^. To better observe the droplet motion process, a red background light was added in the experiment, and a charge coupled device (CCD) camera was used to record this process. Deionized water was used as the experimental object in the experiment. The experiments were repeated four times, and similar results were obtained. The capillary number Ca = *μu*/*γ* (the velocity is small) and the bond number Bo = *gρL*^2^/*γ* (in scale of 10^−2^) were small. Here, *γ* is the surface tension coefficient and *L* was the characteristic length. Therefore, the entire process was treated as quasi-static, and the influence of gravity on the whole process was neglected. In addition, since the experimental process was transitory, and the effect of droplet evaporation was also neglected.

#### 4.1.2 Simulation fundamental

In this paper, an improved model based on Surface Evolver is used to simulate the motion of a droplet from the SPS into the hydrophobic PPS. Surface Evolver is a simulation software used to research fluid stability under the influence of surface tension and other energy and various constraints.^35^ In previous research, a simulation of droplets pinned between the SPS and PPS has been studied by Berthier,^25^ but there are still two shortcomings in the analysis. First, the effect of the side surface was ignored. Second, the impact of CAH on the equilibrium state was not taken into account. To address the first problem, a hydrophilic side surface is added to the model, which coincides with the practical application. To solve the second problem, an approach proposed by Santos and White^36^ is adopted to implement CAH. This approach is achieved by adding a force of friction (the magnitude of which depends on the CAH). In this way, it can be ensured that the contact line only moves when the contact angle is beyond the advancing angle (*α*_a_) or below the receding angle (*α*_r_).

#### 4.1.3 Simulation process

**Fig. 3:**
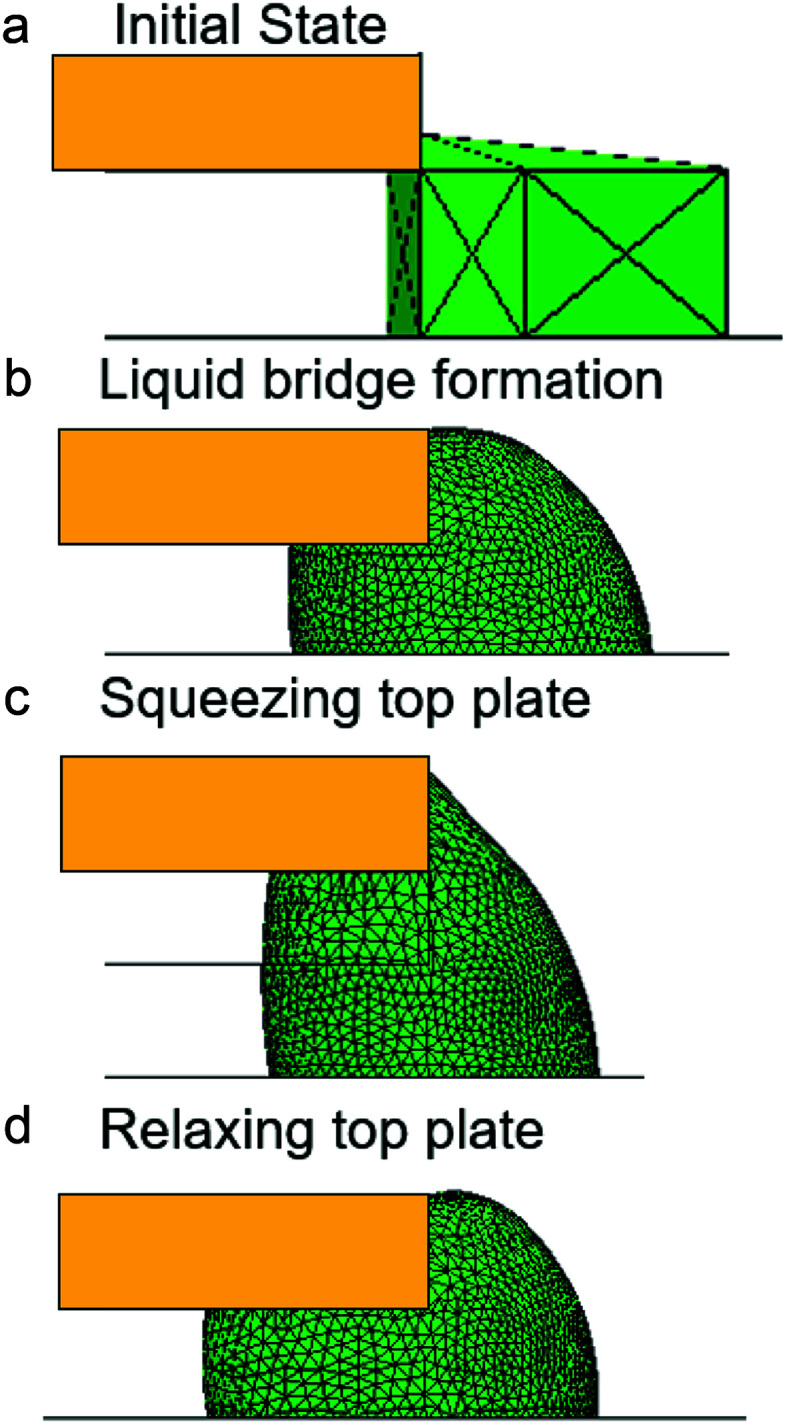
Simulation process of the formation of a liquid bridge and the squeezing and relaxing actions of droplets. (a) Initial state of the droplet before the simulation. (b) Simulation diagram after the formation of the liquid bridge. (c) Simulation diagram after squeezing the liquid bridge. (d) Simulation diagram after relaxing the liquid bridge.

Overall, the whole simulation process has two periods. The first is bridge formation, and the other is motion based on the ratchet-like strategy. In this paper, we pay more attention to the second period, but fundamental information about the bridge forming period is also provided here. Bridge forming (Fig. 3a and b): When the droplet on the bottom plate touched the side surface of the top plate, the liquid expanded into the PPS with *α*_a_ (by capillary force), wetted along the side surface with *θ*_a_, and receded in the SPS with *α*_r_. Therefore, the CAs on the bottom surface in the PPS approached *α*_a_, while those on the bottom surface in the SPS were close to *α*_r_. Ratchet-like motion (Fig. 3b–d): After the equilibrium state was achieved, the top plate was vertically moved to squeeze the bridge step by step in intervals of 0.001 mm. In each step, the bridge shape was reformed with the change of the gap width. After the squeezing process, a similar relaxing process was carried out *via* moving upward step by step in 0.001 mm increments. Simulations were performed using surfaces with different material parameters and control parameters one by one. It's worth emphasizing that the experimental and simulation results were consistent (as shown in Fig. 5–7 and 9).

### 4.2 Process of moving the droplet into the PPS by ratchet-like strategy

From the above experiment, we found that two necessary and sufficient conditions must be satisfied if this inward motion is to be achieved. (1) In the process of relaxing, the tailing edge must first reach the receding angle. (2) In the process of squeezing, the leading edge must first achieve the advancing angle. Based on these two requirements, we have carried on the analysis below.

#### 4.2.1 Analysis of the first requirement

**Fig. 4:**
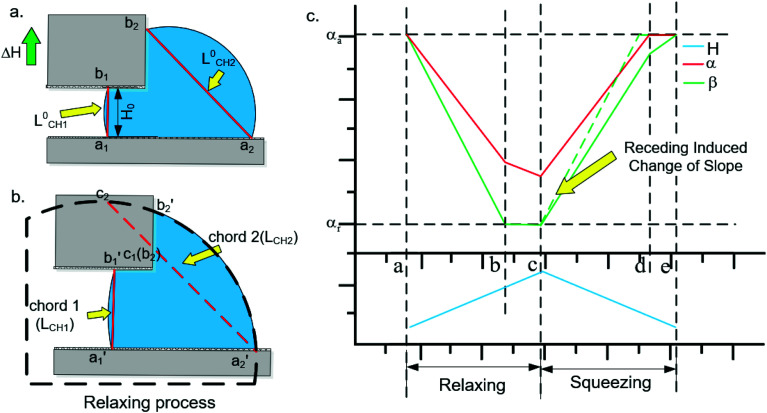
(a) Schematic diagram of the initial state before relaxing. (b) Instantaneous analysis diagram of the contact angles during the relaxing process. (c) Overall relaxing and squeezing process analysis of contact angles *α* and *β*.

First, we define the parameters of the droplets during relaxing action. The parameters in Fig. 4a are consistent with the previous definition. Fig. 4a is the state before the relaxing process. Fig. 4b is the pinned period during the relaxing process. In this figure, the intersection points of the leading edge with the top and bottom plates are 
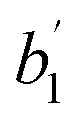
 and 
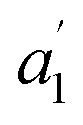
, and the intersection points of the trailing edge with the top and bottom plates are 
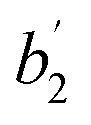
 and 
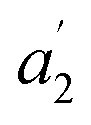
. *c*_1_ in Fig. 4b also indicates the point labelled *b*_2_ in Fig. 4a, so we get 
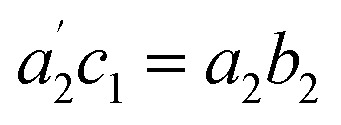
. Point *c*_2_ in Fig. 4b is the intersection point between the extension line of 
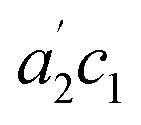
 and the dashed circle (equal curvature circle). The chord 
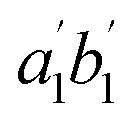
 is marked as chord 1, and the chord 
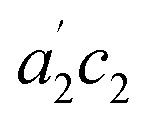
 is marked as chord 2. We assume that *H* = *H*_0_ + Δ*H*. Here, *H*_0_ is the initial width of the gap, and Δ*H* is the rising distance of the top plate. Because the pressure in the droplet is negatively correlated with the width of the gap and the radius of curvature *R*, Δ*H* ∝ *R*, we can assume that *R* = *R*_0_ + *m*Δ*H*, where *m* is the parameter decided by the droplet movement process and *R*_0_ is the initial radius of curvature in the droplet.

Then, we can compare the relationship between *α* and *β* during the relaxing process. According to Fig. 4b, it can be found that 
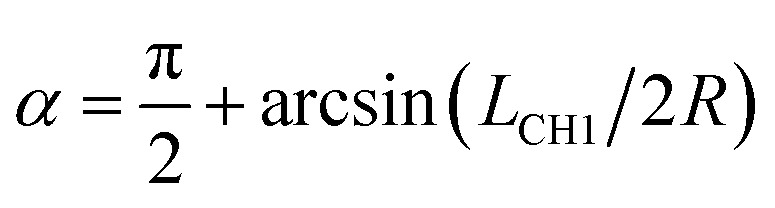
 and 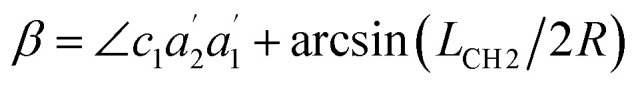. Since 
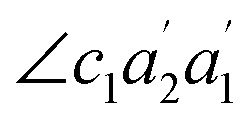
 is a constant value, the comparison between the change rate of *α* and *β* is really the comparison between *L*_CH1_/2*R* and *L*_CH2_/2*R*. The values of *L*_CH1_/2*R* and *L*_CH2_/2*R* can be calculated as:

4 *L*_CH1_/2*R* = (*L*^0^_CH1_ + Δ*H*)/(*R*_0_ + *m*Δ*H*) = (*L*^0^_CH1_ + Δ*H*)/(*R*_0_ + *m*Δ*H*)

5
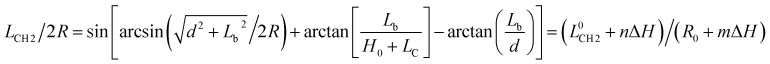
where *n* is a parameter indicating the change rate of *L*_CH2_ and *d* = *H*_0_ + *L*_C_ + Δ*H*. *L*^0^_CH1_ and *L*^0^_CH2_ are the initial lengths of chord 1 and chord 2, and *H*_0_ is the initial width of the gap. Since the rate of change of *R* is much greater than the rate of change of Δ*H*, *m* ≫ 1. Then, *L*_CH1_/2*R* ≈ *L*^0^_CH1_/(*R*_0_ + *m*Δ*H*) and *L*_CH2_/2*R* ≈ *L*^0^_CH2_/(*R*_0_ + *m*Δ*H*). So, we can get:6
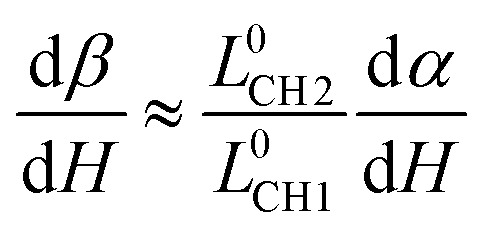


Therefore, since *L*^0^_CH2_ > *L*^0^_CH1_ (according Fig. 4a), the change rate of *β* with Δ*H* is larger than the change rate of *α* with Δ*H*. In addition, since the initial value of *β* is smaller than *α*, during the relaxation process, *β* is always smaller than *α*. In other words, the first requirement can be satisfied.

#### 4.2.2 Analysis of the second requirement

Afterward, the relationship between contact angles *α* and *β* during the squeezing process was also analysed. Compared to the relaxing process, the parameters *L*_a_, *L*_b_ are changed (*L*_a_ increase, *L*_b_ decrease), but *L*_C_ maintains a similar value (sliding along the side surface is not considered here). According to eqn (4), *L*_a_ has little effect on the value of *L*_CH1_, which means that the change rate of *α* during relaxing and squeezing processes is similar. Conversely, according to eqn (5), the smaller is *L*_b_, the smaller are change rates of the term 
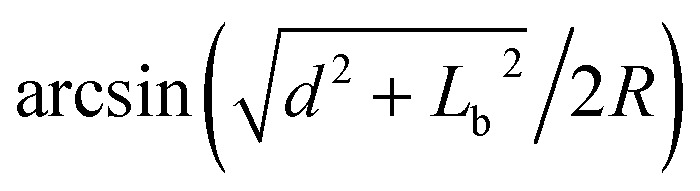
 and the term arctan [*L*_b_/(*H*_0_ + L_C_)] − arctan(*L*_b_/*d*), the smaller is the change rate of the length of chord 2, and the smaller is the change rate of contact angle *β*. Thus, it can be seen that the change of contact angle *β* during the squeezing process is slower than that in the relaxing process. Therefore, compared with *β* rapidly reaching the receding angle during the relaxing process, it is much more difficult for *β* to reach the advancing angle. This leads to satisfaction of the third requirement.

#### 4.2.3 Overall process analysis

By combining the above analyses, we obtain a schematic diagram of the changes of the contact angles during the whole relaxing and squeezing process, as shown in Fig. 4c. The red line is the prediction line of CA *α*, the green line is the prediction line of CA *β*, and the blue line is the theoretical curve of the width of the gap. (a)–(c) is the relaxing process, and (c)–(e) is the squeezing process. First, in the process of (a) and (b), *β* approaches *α*_r_ faster than *α*. Second, in the process of (b) and (c), the receding movement occurs first in SPS, and due to this displacement, the *α* reduction also slows. Third, in the process of (c) and (d), compared with the relaxing process, the change rate of *α* is the same, but the change rate of *β* decreases (the green dotted line changing to a green solid line). So, when the slope is low enough, *α* will reach *α*_a_ first. Finally, in the process of (d) and (e), advancing movement occurs in the PPS first, and due to this displacement, the decrease of *β* also slows. On the basis of these four processes, we can predict the changes of the contact angles throughout the whole ratchet motion. Through this prediction, we find that the droplet can conduct directional motion. This illustrates that the main cause of droplet movement through the ratchet strategy is the asymmetric change of CAs *α* and *β* induced by the asymmetric structure.

#### 4.2.4 Verification by simulations and experiments

**Fig. 5:**
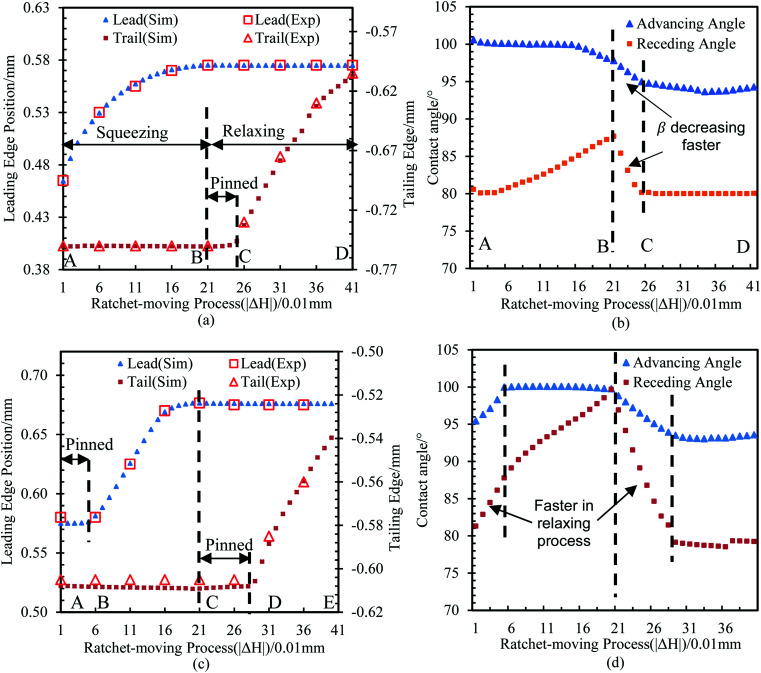
First and second ratchet cycle used to illustrate the ratchet-like process. (a) Leading and trailing edges (b) the CAs on the bottom surface *versus* the ratchet-moving process in the first ratchet cycle. (c) Leading and trailing edges. (d) The CAs on the bottom surface *versus* the ratchet-moving process in the second ratchet cycle.

Finally, we verified the above analysis *via* experiments and simulations. The result of the inward motion in the first two cycles after bridge formation is shown in Fig. 5. The bridge is squeezed and relaxed from *H*_max_ = 0.5 mm to *H*_min_ = 0.3 mm (marked as the reference condition, which is also point D in Fig. 2b).

In the first cycle, three processes were performed, as shown in Fig. 5a. First, the bridge started to move toward the PPS directly, but the trailing edge remained pinned (A and B in Fig. 5a). Second, the bridge was relaxed. At this stage, since the contact lines were pinned by CAH, no obvious movement of the liquid bridge was observed (from B and C in Fig. 5a). Meanwhile, *α* and *β* decreased, and according to the period B and C in Fig. 5b, *β* (about 140° mm^−1^) had a faster decreasing speed than that of *α* (about 50° mm^−1^). Third, when *β* quickly reached the receding angle *α*_r_, the trailing edge started to recede towards the PPS (C and D in Fig. 5a), while the leading edge was pinned.

In the second cycle, four processes were performed, as shown in Fig. 5c. Compared with the first cycle, we found that a pinned period occurred at the beginning of the squeezing process. At this stage, the contact line remained pinned while *α* and *β* increased. Since *α* and *β* were within the CAH interval, no inward motion was observed (the leading edge and trailing edge remained stationary in Fig. 5c). The corresponding CA change processes are shown in Fig. 5d, and it can also be found that *β* decreased (about 250° mm^−1^) much faster than did *α* (about 69° mm^−1^) during the relaxing process, in addition to *β* having a faster decreasing speed (about 250° mm^−1^) in the relaxing process compared to the increasing speed (about 95° mm^−1^) in the squeezing process. All of these findings are consistent with our previous analysis.

The above example validates our theoretical analysis indicating that the main cause of droplet movement through the ratchet strategy is the asymmetric change of CAs *α* and *β* during the squeezing and relaxing process, which has never been mentioned in previous studies. In addition to exploring the reasons why this strategy can be implemented, we have also optimized this method to achieve a higher transport efficiency. We found that the critical factors determining the efficiency of the inward motion mainly include two characteristics.

(a) The length of the “pinned period”: The range of |Δ*H*|, which is used to overcome the contact line pinning at the beginning of relaxing or squeezing, is an important factor affecting the efficiency.

(b) The “change rate” of Δ*X versus* |Δ*H*| (|d*X*/d*H*|): when the bridge begins to move, the movement efficiency is determined by the change rate of Δ*X versus* |Δ*H*|. A larger |d*X*/d*H*| will cause a larger distance of horizontal motion with the same change of |Δ*H*|, which also means a higher efficiency.

To achieve better inward motion performance of the bridge, the effects of various parameters are discussed and optimized in the following subsections.

### 4.3 Effect of the parameters on Δ*X*

#### 4.3.1 Effect of the control parameters (*H*_0_, Δ*H*) on ΔX

In this subsection, we discuss the method of optimizing the motion by varying the control parameters according to the corresponding simulation and experimental results.

**Fig. 6:**
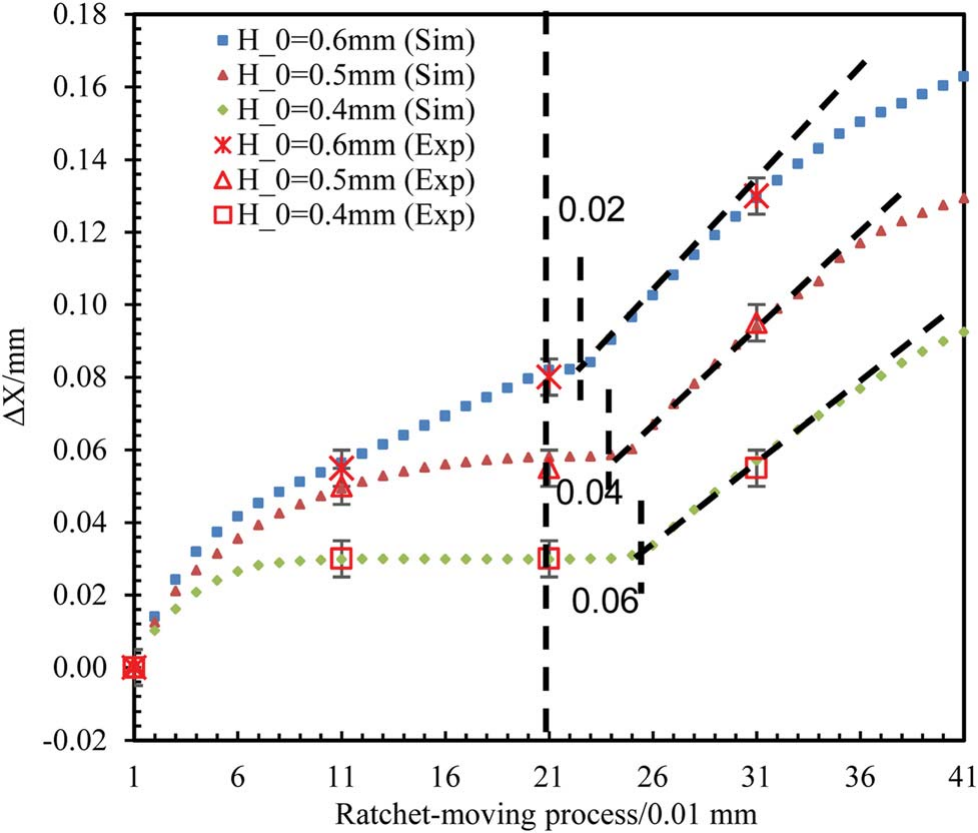
Motion of the bridge with the ratchet process under the reference conditions but with three different values of *H*_0_ at 0.4 mm, 0.5 mm and 0.6 mm; thus, the results are from both simulations and experiments.

The effect of *H*_0_ is first addressed. The motion of the bridge under the reference conditions and with three different values of *H*_0_ (varying from 0.4 mm to 0.6 mm) is demonstrated. The results are shown in Fig. 6, where it can be found that varying *H*_0_ has two main effects on the inward movement. A larger *H*_0_ (0.6 mm, 0.5 mm and 0.4 mm, respectively) corresponds to a smaller pinned period (0.02 mm, 0.04 mm and 0.06 mm, respectively) and a slightly larger |d*X*/d*H*| (0.50, 0.48 and 0.44, respectively). These observations can be explained by the pressure difference between the PPS and SPS demonstrated in Section 3.1. As shown in Fig. 2b, points C, D, E, and F represent the situation when *H*_0_ = 0.6 mm, *H*_0_ = 0.5 mm, *H*_0_ = 0.4 mm and Δ*P* = 0. Since CF > DF > EF, a stronger tendency (larger pressure) of inward motion will be caused when H_0_ is larger, and then a smaller pinned period and a larger value of |d*X*/d*H*| will be produced.

**Fig. 7:**
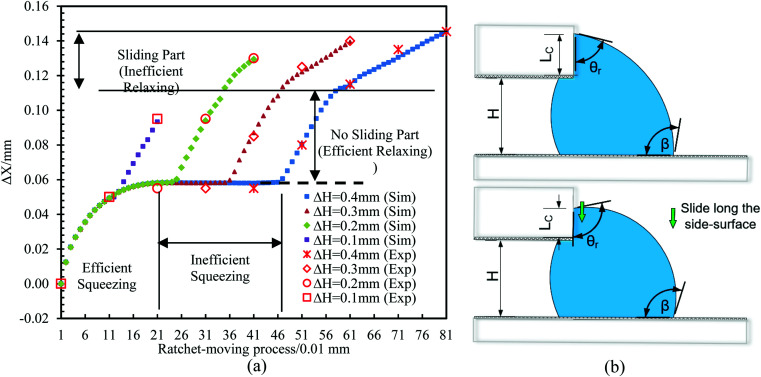
Motion of the bridge with the ratchet process under the reference conditions but with three different Δ*H* at 0.1 mm, 0.2 mm, 0.3 mm and 0.4 mm. The results are from simulations and experiments.

The effects of Δ*H* are also addressed. The motion of the bridge under the reference conditions and with four different values of Δ*H* (from 0.1 mm to 0.4 mm) is demonstrated. The results are shown in Fig. 6a, where it can be found that a larger ΔH can increase the inward movement, but when Δ*H* > 0.2 mm, there are some “inefficient” processes. This is mainly caused by two factors. First, during the squeezing process, according to the pressure analysis in Section 3.1, the force pressure on the droplet will gradually change from inward to outward, which will hinder or even stop the droplet's inward movement into the PPS (shown as the “Inefficient squeezing” part in Fig. 7a). Second, during the relaxing process, the droplets slide on the side surface due to excessive lifting of the top plate (Δ*H* > 0.2 mm, as shown in Fig. 7b). This downward sliding motion prevents the droplet from moving backward in the SPS. This greatly reduces the efficiency of the droplets' entry into the PPS (“Sliding part” in Fig. 7a). Thus, it is crucial to find the appropriate Δ*H* to achieve efficient droplet movement.

#### 4.3.2 Effect of the thickness of the top plate on Δ*X*

**Fig. 8:**
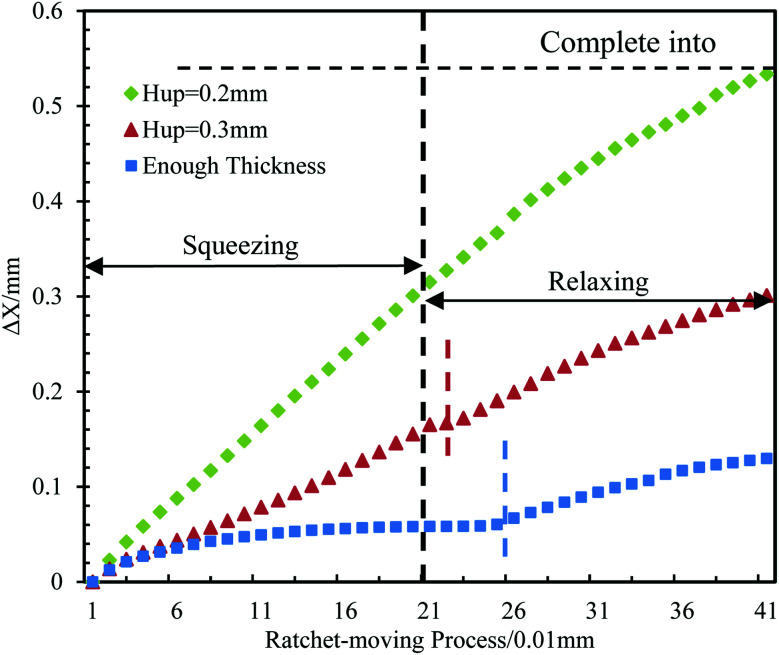
Simulation of the value of Δ*X versus* |Δ*H*| for the same situation as shown in Fig. 3 but with three different values of *H*_up_ at 0.2 mm, 0.3 mm and no limit.

Because a greater *θ*_a_ can promote inward motion (as explained in Section 3.1), we estimate that a thinner top plate (has a similar function of larger *θ*_a_) will produce a better drive performance. We verify this idea through simulations. As shown in Fig. 8, the value of Δ*X versus* |Δ*H*| is plotted for the same situation as used for the reference point but with three different values of *H*_UP_. It can be found that varying *H*_UP_ has two main effects on the inward movement. A smaller *H*_UP_ (0.2 mm, 0.3 mm and no limit, respectively) corresponds to a smaller pinned period (0.00 mm, 0.02 mm and 0.05 mm, respectively) and a larger |d*X*/d*H*| (1.25, 0.75 and 0.31, respectively). Therefore, the results prove our idea that a better performance can be achieved with a thinner top plate during the whole ratchet cycle.

**Fig. 9:**
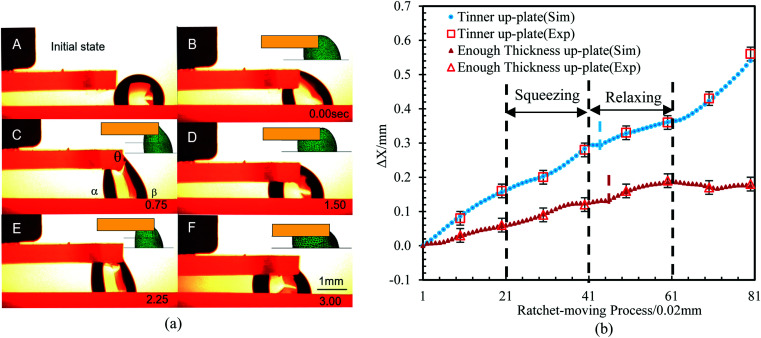
Experiments using the optimized ratchet cycle with different top plate thicknesses. (a) Time sequence illustrating the water droplet transport generated by the relaxing and squeezing cycles of the combined structure. During the squeezing process, the leading contact line advances towards the PPS, and during the relaxing process, the trailing contact line recedes away from the SPS. The profiles of the experiments and simulations are consistent. Two ratchet cycles are required for the droplets to fully enter the PPS. (b) Comparisons of the moving efficiency of different top plate thicknesses based on the simulations and experiments.

### 4.4 Optimizing the ratchet cycle by adjusting the parameters

Finally, experiments with optimized parameters were carried out (*V* = 1.5 μL and *H*_UP_ = 0.6 mm) to verify our analysis. In these experiments, the top/bottom surfaces were hydrophobic-treated (*α*_a_ ≈ 100°, *α*_CAH_ ≈ 20°), while the side surface was untreated (*θ*_a_ ≈ 75°, *θ*_CAH_ ≈ 30°). As shown in Fig. 9a, when we chose an appropriate *H* range (0.5–0.9 mm) so that the droplets could move directionally through ratchet-like motion with high efficiency, two ratchet cycles were required for the droplets to fully enter the PPS. The simulations were consistent with the experiments (the profiles of the simulation and experimental results were consistent). To highlight the better performance of the thin top plate, the moving distance (Δ*X*) of the liquid bridge with a limited (0.6 mm) and a sufficient top plate thickness were compared through simulations and experiments. The results are shown in Fig. 9b, where it can be found that a smaller *H*_UP_ (0.6 mm and no limit, respectively) corresponds to a smaller pinned period (0.03 mm and 0.06 mm, respectively) and a larger |d*X*/d*H*| (0.50 and 0.31, respectively), which is consistent with our analysis above.

## 5. Conclusions

In summary, experimental and numerical approaches, as well as a theoretical analysis, were employed to study the process of a droplet moving into hydrophobic parallel plates. A ratchet-like strategy was used for the first time to transport the droplets into the hydrophobic parallel plates. In this work, from a static point of view, we analyzed the force applied to the droplet. Afterward, we provided the reason that why this strategy can achieve droplet entry into the PPS from the theoretical point of view, which was verified by experiments and simulations. Then, the functions of the control parameters (*H*_0_, Δ*H*) and the thickness of the top plate regarding the efficiency of the movement were researched. Finally, an optimized ratchet cycle was carried out to verify our analysis.

Compared with previous studies,^25,30,31,37,38^ the present strategy provides the following new advances.

(a) A new method of transporting droplets into hydrophobic parallel plates was proposed. Since only mechanical forces are required, this means that no electric fields or other methods that might affect biological or chemical substances are needed, making the method safer for many biochemical applications.

(b) We creatively put forward that the asymmetric change of the CAs induced by the asymmetric structure is the fundamental cause of this kind of motion.

(c) The length of the chord is used for the first time to express the change rate of the contact angle, from which we can easily determine whether the directional motion of the liquid bridge can be achieved by such a ratchet or ratchet-like strategy.

## Conflicts of interest

There are no conflicts to declare.

## Supplementary Material

## References

[cit1] Shang L., Cheng Y., Zhao Y. (2017). Emerging Droplet Microfluidics. Chem. Rev..

[cit2] Liu C., Sun J., Li J., Xiang C., Che L., Wang Z., Zhou X. (2017). Long-range spontaneous droplet self-propulsion on wettability gradient surfaces. Sci. Rep..

[cit3] Lai Y. H., Yang J. T., Shieh D. B. (2010). A microchip fabricated with a vapor-diffusion self-assembled-monolayer method to transport droplets across superhydrophobic to hydrophilic surfaces. Lab Chip.

[cit4] Brochard F. (1995). Motions of droplets on solid surfaces induced by chemical or thermal gradients. Br. J. Clin. Psychol..

[cit5] Linke H., Alemán B. J., Melling L. D., Taormina M. J., Francis M. J., Dow-Hygelund C. C., Narayanan V., Taylor R. P., Stout A. (2006). Self-propelled Leidenfrost droplets. Phys. Rev. Lett..

[cit6] Li J., Hou Y., Liu Y., Hao C., Li M., Chaudhury M. K., Yao S., Wang Z. (2016). Directional transport of high-temperature Janus droplets mediated by structural topography. Nat. Phys..

[cit7] Wu J., Ma R., Wang Z., Yao S. (2011). Do droplets always move following the wettability gradient?. Appl. Phys. Lett..

[cit8] Ng A. H., Li B. B., Chamberlain M. D., Wheeler A. R. (2015). Digital Microfluidic Cell Culture. Annu. Rev. Biomed. Eng..

[cit9] Ng A. H., Lee M., Choi K., Fischer A. T., Robinson J. M., Wheeler A. R. (2015). Digital microfluidic platform for the detection of rubella infection and immunity: a proof of concept. Clin. Chem..

[cit10] Shen H.-H., Fan S.-K., Kim C.-J., Yao D.-J. (2014). EWOD microfluidic systems for biomedical applications. Microfluid. Nanofluid..

[cit11] Peng C., Zhang Z., Kim C. J., Ju Y. S. (2014). EWOD (electrowetting on dielectric) digital microfluidics powered by finger actuation. Lab Chip.

[cit12] Banno T., Kuroha R., Toyota T. (2012). pH-Sensitive self-propelled motion of oil droplets in the presence of cationic surfactants containing hydrolyzable ester linkages. Langmuir.

[cit13] Sartori P., Quagliati D., Varagnolo S., Pierno M., Mistura G., Magaletti F., Casciola C. M. (2015). Drop motion induced by vertical vibrations. New J. Phys..

[cit14] Mettu S., Chaudhury M. K. (2011). Motion of liquid drops on surfaces induced by asymmetric vibration: role of contact angle hysteresis. Langmuir.

[cit15] John K., Thiele U. (2010). Self-ratcheting Stokes drops driven by oblique vibrations. Phys. Rev. Lett..

[cit16] Dong L., Chaudhury A., Chaudhury M. K. (2006). Lateral vibration of a water drop and its motion on a vibrating surface. Eur. Phys. J. E: Soft Matter Biol. Phys..

[cit17] Brunet P., Eggers J., Deegan R. D. (2009). Motion of a drop driven by substrate vibrations. Eur. Phys. J.: Spec. Top..

[cit18] Brunet P., Eggers J., Deegan R. D. (2007). Vibration-induced climbing of drops. Phys. Rev. Lett..

[cit19] Sadeghi S., Ding H., Shah G. J., Chen S., Keng P. Y., Kim C. J., van Dam R. M. (2012). On chip droplet characterization:
a practical, high-sensitivity measurement of droplet impedance in digital microfluidics. Anal. Chem..

[cit20] Hadwen B., Broder G. R., Morganti D., Jacobs A., Brown C., Hector J. R., Kubota Y., Morgan H. (2012). Programmable large area digital microfluidic array with integrated droplet sensing for bioassays. Lab Chip.

[cit21] Samiei E., Tabrizian M., Hoorfar M. (2016). A review of digital microfluidics as portable platforms for lab-on a-chip applications. Lab Chip.

[cit22] Wang G., Teng D., Lai Y. T., Lu Y. W., Ho Y., Lee C. Y. (2014). Field-programmable lab-on-a-chip based on microelectrode dot array architecture. IET Nanobiotechnol..

[cit23] Paxson A. T., Varanasi K. K. (2013). Self-similarity of contact line depinning from textured surfaces. Nat. Commun..

[cit24] Heng X., Luo C. (2014). Bioinspired Plate-Based Fog Collectors. ACS Appl. Mater. Interfaces.

[cit25] Berthier J. , Microdrops and Digital Microfluidics, 2008

[cit26] Moon I., Kim J. (2006). Using EWOD (electrowetting-on-dielectric) actuation in a micro conveyor system. Sens. Actuators, A.

[cit27] Daniel S., Sircar S., Gliem J., Chaudhury M. K. (2004). Ratcheting motion of liquid drops on gradient surfaces. Langmuir.

[cit28] Prakash M., Quere D., Bush J. W. (2008). Surface tension transport of prey by feeding shorebirds: the capillary ratchet. Science.

[cit29] Concus P., Finn R. (1998). Discontinuous behavior of liquids between parallel and tilted plates. Phys. Fluids.

[cit30] Ataei M., Tang T., Amirfazli A. (2017). Motion of a liquid bridge between nonparallel surfaces. J. Colloid Interface Sci..

[cit31] Huang Y., Hu L., Chen W., Fu X., Ruan X., Xie H. (2018). Directional Transport of a Liquid Drop between Parallel-Nonparallel Combinative Plates. Langmuir.

[cit32] Heng X., Luo C. (2015). Liquid drop runs upward between two nonparallel plates. Langmuir.

[cit33] Luo C., Heng X., Xiang M. (2014). Behavior of a liquid drop between two nonparallel plates. Langmuir.

[cit34] Wang L., Wu H., Wang F. (2016). Efficient transport of droplet sandwiched between saw-tooth plates. J. Colloid Interface Sci..

[cit35] Collicott S. H., Weislogel M. M. (2004). Computing existence and stability of capillary surfaces using surface evolver. AIAA J..

[cit36] Santos M. J., White J. A. (2011). Theory and Simulation of Angular Hysteresis on Planar Surfaces. Langmuir.

[cit37] Wang W., Jones T. B. (2015). Moving droplets between closed and open microfluidic systems. Lab Chip.

[cit38] Ataei M., Chen H., Amirfazli A. (2017). Behavior of a Liquid Bridge between Nonparallel Hydrophobic Surfaces. Langmuir.

